# Endostatin attenuates heart failure via inhibiting reactive oxygen species in myocardial infarction rats

**DOI:** 10.1042/BSR20200787

**Published:** 2021-06-29

**Authors:** Xuguang Xu, Tingbo Jiang, Yong Li, Liusha Kong

**Affiliations:** 1 Department of Cardiology, The First Affiliated Hospital of Soochow University, Suzhou, China; 2 Department of Cardiology, The Affiliated Hospital of Xuzhou Medical University, Xuzhou, China; 3 Department of Cardiology, The First Affiliated Hospital of Nanjing Medical University, Nanjing, China; 4 Department of Nephrology, The Affiliated Hospital of Xuzhou Medical University, Xuzhou, China

**Keywords:** cardiac function, endostatin, heart failure, myocardial infarction, oxidative stress

## Abstract

The purpose of the present study was to evaluate whether endostatin overexpression could improve cardiac function, hemodynamics, and fibrosis in heart failure (HF) via inhibiting reactive oxygen species (ROS). The HF models were established by inducing ischemia myocardial infarction (MI) through ligation of the left anterior descending (LAD) artery in Sprague–Dawley (SD) rats. Endostatin level in serum was increased in MI rats. The decrease in cardiac function and hemodynamics in MI rats were enhanced by endostatin overexpression. Endostatin overexpression inhibited the increase in collagen I, collagen III, α-smooth muscle actin (α-SMA), connective tissue growth factor (CTGF), matrix metalloproteinase (MMP)-2 and MMP9 in the hearts of MI rats. MI-induced cardiac hypertrophy was reduced by endostatin overexpression. The increased levels of malondialdehyde (MDA), superoxide anions, the promoted NAD(P)H oxidase (Nox) activity, and the reduced superoxide dismutase (SOD) activity in MI rats were reversed by endostatin overexpression. Nox4 overexpression inhibited the cardiac protective effects of endostatin. These results demonstrated that endostatin improved cardiac dysfunction and hemodynamics, and attenuated cardiac fibrosis and hypertrophy via inhibiting oxidative stress in MI-induced HF rats.

## Introduction

Heart failure (HF) is showing an increasing prevalence [1]. The risk of HF can be elevated by myocardial infarction (MI) [2], in which cardiac remodeling occurs. Cardiac remodeling is characterized by increasing fibrosis, and accumulation of collagen type I, collagen type III, α-smooth muscle actin (α-SMA), connective tissue growth factor (CTGF), matrix metalloproteinase (MMP) 2 and MMP9 [3–6]. Unfortunately, the pathophysiological mechanisms in the failing heart remain largely unknown and the only curative end stage therapy is transplantation.

Endostatin, a C-terminal fragment of collagen XVIII located in the vascular basement membrane, can be cleaved by various proteases including cathepsins, MMPs, or elastase [7–9]. Endostatin participates in the healing process after MI by activating myofibroblasts [10]. The expression level of endostatin in heart tissues has been reported to increase in the experimental cardiac disease models, such as MI [11,12] and pressure overload-induced cardiac hypertrophy [13,14]. Higher serum endostatin is associated with left ventricular (LV) dysfunction and an increased HF risk, but further experimental studies are needed to investigate the role of endostatin in the development of HF [15].

Reactive oxygen species (ROS) generated during cellular aerobic respiration and metabolism are implicated in cardiovascular diseases [16,17]. Peripheral blood mononuclear cells isolated from chronic HF patients show a mitochondrial population consisting of damaged and less functional organelles, which is responsible for higher superoxide anion production [18]. Endostatin treatment significantly decreases prostate cancer cell proliferation through up-regulation of manganese superoxide dismutase (SOD) and the reduced glutathione [19]. Endostatin stimulates cell proliferation, migration, and wound-induced migration of adult rat cardiac fibroblasts at least partly through the ROS-dependent activation of protein kinase B [20]. However, whether endostatin attenuates HF via inhibiting ROS is not well understood.

We addressed this in the present study by investigating whether endostatin overexpression could reverse the cardiac dysfunction, fibrosis, and decreased cardiac hemodynamics in the hearts of MI rats. Furthermore, we tested whether the cardiac protective effects of endostatin in MI rats could be achieved via inhibiting the ROS level.

## Materials and methods

### Animals

The experiments were carried out using 160–180 g male Sprague–Dawley (SD) rats (Vital River Biological Co., Ltd, Beijing, China) in the Animal Core Facility of Xuzhou Medical University. The rats were kept in a temperature-controlled room on a 12-h light–dark cycle with free access to standard chow and tap water. All procedures were approved by the Experimental Animal Care and Use Committee of Xuzhou Medical University, and conducted in accordance with the Guide for the Care and Use of Laboratory Animals (NIH publication number 85-23, revised 1996).

### MI model

In the present study, MI in rats was induced by coronary artery ligation with sterile techniques as previously reported [21] because it is a much more solid method to induce HF than left circumflex artery (LCX) ligation [22]. Briefly, the rats were anesthetized with sodium pentobarbital (50 mg.kg^−1^, i.p.) and randomly subjected to the ligation of the left anterior descending (LAD) coronary artery or the sham surgery. The heart was exposed through the left intercostal thoracotomy, and the left coronary artery was looped by a single nylon suture. Finally, the heart was quickly repositioned into the chest. The sham-operated rats were treated the same way as the coronary-ligation rats except that their coronary arteries were not ligated.

### Echocardiography

Transthoracic echocardiography was performed under isoflurane anesthesia (2.5%) using an ultrasound system (VisualSonics, Toronto, Canada) with a 21-MHz probe. The LV weight, LV end-diastolic diameter (LVEDD) and end-systolic diameter (LVESD), and LV volumes in diastole (LVVD) and systole (LVVS) were measured. The LV ejection fraction (EF) and fractional shortening (FS) were calculated. Measurements over three consecutive cardiac cycles were averaged.

### Hemodynamic monitoring

A conductance micromanometer catheter (1.4F, Millar Instruments, TX, U.S.A.) was inserted via the right carotid artery across the aortic valve and into the LV chamber of the anesthetized rat with isoflurane (2.5%). The maximum of the first differentiation of LV pressure (LV +dp/dt) and decline (LV −dp/dt), LV systolic pressure, and LV end-diastolic pressure (LVEDP) were obtained using a PowerLab data acquisition system (AD Instruments, Sydney, Australia).

### Endostatin level determination by ELISA

Rats were anesthetized under isoflurane (2.5%). Blood was collected from heart, and then rats were killed by perfusion with PBS. The heart was removed immediately. Endostatin levels in the serum and heart were determined with an ELISA kit (USCN Business Co., Ltd., Wuhan, China) according to the manufacturer’s instructions. Briefly, 50 µl standard or sample and 50 µl prepared Detection Reagent A was added to each well and incubated for 1 h at 37°C. Next, 100 µl prepared Detection Reagent B was added and incubated for 30 min at 37°C. Then, 90 µl substrate solution was added into each well and incubated for 10–20 min at 37°C. Finally, 50 µl stop solution was added and read at 450 nm immediately.

### Western blotting

The heart samples were sonicated in RIPA lysis buffer (Nanjing BioChannel Biotechnology Co., Ltd., Nanjing, China) and homogenized. The debris was removed by centrifugation at 12000×***g*** for 10 min at 4°C and the supernatant was collected. Subsequently, ∼30–40 μg protein was separated by 8% gel electrophoresis, and transferred to PVDF membrane. The membrane was blocked with 5% skimmed milk powder at room temperature for 1 h and probed with primary antibody overnight at 4°C against NAD(P)H oxidase (Nox4; 1:1000, Abcam, MA, U.S.A.). Then, horseradish peroxidase-conjugated goat anti-rabbit secondary antibody (1:10000, Abcam) was added and incubated at room temperature for 1 h and GAPDH (1:10000, Abcam) was used as an internal control. The bands were visualized via ECL (Beyotime, Shanghai, China). Images were analyzed using Image-Pro Plus software (CAD/CAM Services, Inc.).

### Masson’s trichrome staining

The cardiac sections (5 µm) were examined by Masson’s trichrome staining (Service Biological Technology Co., Ltd, Wuhan, China) to determine the extent of fibrosis according to the manufacturer’s instructions. Tissue sections from rat hearts were observed under light microscopy (Carl Zeiss GmbH, Oberkochen, Germany). Images were analyzed using Image-Pro Plus software (Media Cybernetics, Inc., MD, U.S.A.).

### Wheat germ agglutinin staining

Cardiac sections were stained using FITC-conjugated wheat germ agglutinin (WGA; Invitrogen Inc., CA, U.S.A.) to measure the cross-sectional area of cardiomyocytes. Three to five random fields were selected from each of five sections from each rats for observation under a confocal microscope (Carl Zeiss GmbH, Oberkochen, Germany) and analyzed with Zeiss software.

### Endostatin or NADPH oxidases 4 overexpression

Recombinant adenoviral vectors harboring endostatin (Ad-Endostatin), Nox4 (Ad-Nox4) or enhanced green fluorescent protein (Ad-GFP) were made by Genechem Company Ltd. (Shanghai, China). Adenovirus (1 × 10^10^ TU/ml) was injected into the rat via the tail vein at the same time of MI surgical operation.

### Malondialdehyde level in the heart

The LV samples were homogenized in lysis buffer (Thermo Fisher Scientific, MA, U.S.A.). The malondialdehyde (MDA) level in the heart was determined using the ELISA kit (USCN Business Co., Ltd., Wuhan, China) following the manufacturer’s instructions.

### SOD activity level

The LV samples were collected and homogenated. SOD measurement was performed according to the manufacturer’s instructions (Jiancheng Bioengineering Institute, Nanjing, China) using a microplate reader (BioTek, VT, U.S.A.).

### Measurement of Nox activity

The Nox activity in the heart was measured by enhanced lucigenin chemiluminescence. Briefly, NAD(P)H (100 μM) was added to the media as a substrate to react with Nox and generate superoxide anions. The light emission produced by the reaction of lucigenin (5 μM) with superoxide anions was measured with a microplate reader (BioTek, VT, U.S.A.) once every minute for 10 min. The value representing the No activity were expressed as the mean light units (MLUs) per minute per milligram of protein.

### Measurement of superoxide anions

The superoxide anions level in the heart was determined by lucigenin-derived chemiluminescence. Briefly, the reaction with superoxide anions was started by adding dark-adapted lucigenin (5 μM) to each sample to cause photon emission, which was measured with a microplate reader (BioTek, VT, U.S.A.) once every minute for 10 min. The value representing the superoxide anions level was expressed as the MLU per minute per milligram of protein.

### Statistical analyses

Data were presented as the mean ± standard error of the mean (SE) and analyzed using GraphPad Prism 7.0 (GraphPad software Inc., CA, U.S.A.). Statistics were completed using one-way or two-way ANOVA, followed by Bonferroni test for post hoc analysis when multiple comparisons were made. A two-tailed *P*-value <0.05 was considered statistically significant.

## Results

### Endostatin expression

**Figure 1:**
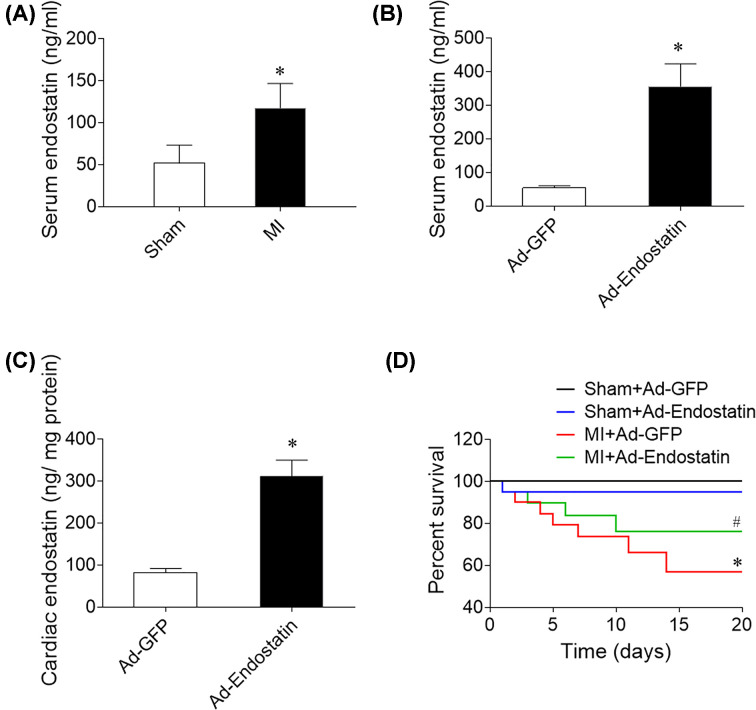
Endostatin overexpression increased survival rate of MI rats (**A**) Endostatin level was increased in the serum of MI rats. (**B**) Serum endostatin level was increased in Ad-Endostatin treatment rats. (**C**) Cardiac endostatin level was increased in Ad-Endostatin treatment rats. (**D**) Endostatin overexpression increased survival rate of MI rats. The results are expressed as mean ± SEM. *n*=12. *P<0.05 versus the Sham group; ^#^*P*<0.05 versus the MI+Ad-GFP group.

Endostatin levels increased in the serum of MI rats (Figure 1A). The levels of endostatin in the serum (Figure 1B) and heart (Figure 1C) were increased in the rat treatment with Ad-Endostatin.

### Effects of endostatin overexpression on survival of MI rats

The survival rate was reduced in MI rats compared with the sham surgery group. Endostatin overexpression increased the survival rate of MI rats (Figure 1B).

### Effects of endostatin overexpression on cardiac dysfunction in MI rats

**Figure 2:**
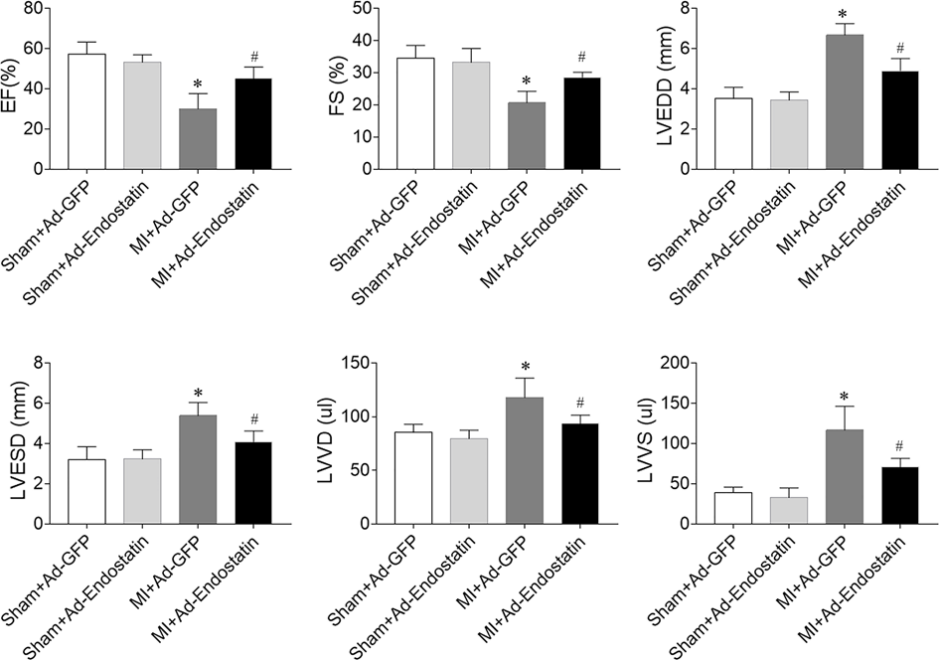
Endostatin improved cardiac dysfunction in MI rats The decreases in LV EF and FS, and the increases in LVEDD, LVEDS, LVVD, and LVVS in MI rats were reversed by endostatin overexpression. The results are expressed as mean ± SEM. *n*=8. *P<0.05 versus the Sham+Ad-GFP group; ^#^*P*<0.05 versus the MI+Ad-GFP group.

LVEF is superior to dp/dt_max_ in the evaluation of HF [23]. The MI-induced reduction in EF (%) and FS (%) was reversed by endostatin overexpression. Endostatin overexpression inhibited the MI-induced increases in LVEDD, LVESD, LVVD, and LVVS (Figure 2).

### Effects of endostatin overexpression on cardiac hemodynamics

**Figure 3:**
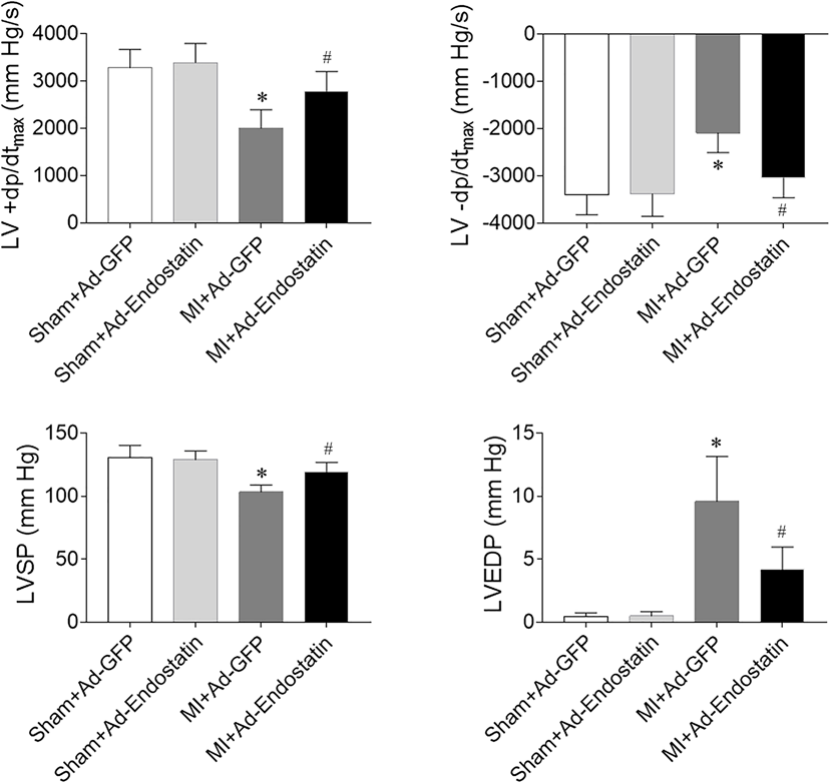
Endostatin improved the impaired cardiac hemodynamics in MI rats The decreases in the maximum of the first differentiation of LV pressure (LV ±dp/dt_max_), LVSP, and the increase in LVEDP in MI rats were reversed by endostatin overexpression. The results are expressed as mean ± SEM. *n*=8. *P<0.05 versus the Sham+Ad-GFP group; ^#^*P*<0.05 versus the MI+Ad-GFP group.

LV ±dp/dt_max_ and LV systolic pressure (LVSP) were decreased in MI rats, which was reversed by endostatin overexpression. The MI-induced reduction in LVEDP was inhibited by endostatin overexpression (Figure 3).

### Effects of endostatin overexpression on cardiac remodeling

**Figure 4:**
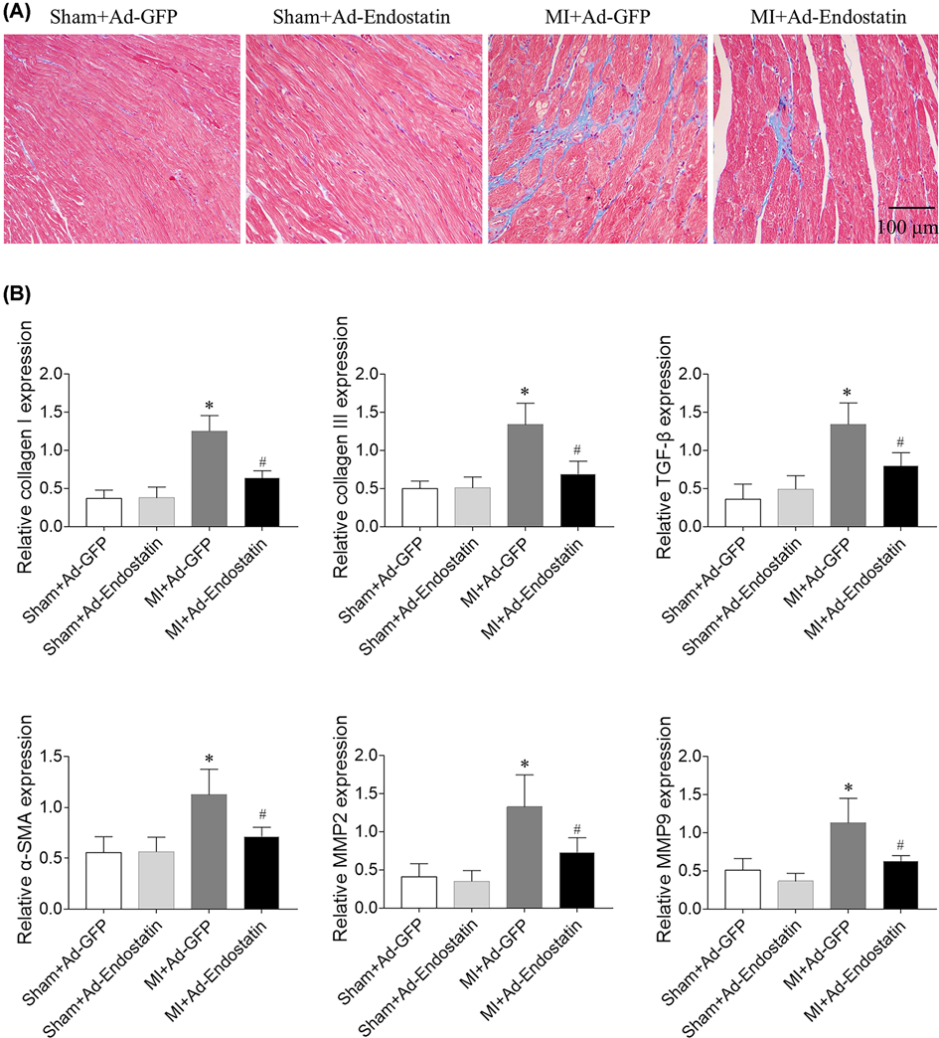
Endostatin attenuated cardiac fibrosis in MI rats (**A**) Endostatin overexpression attenuated heart fibrosis of MI rats as revealed by Masson’s staining. (**B**) The increased expressions of collagen I, collagen III, α-SMA, CTGF, MMP-2 and MMP9 were inhibited by endostatin overexpression. The results are expressed as mean ± SEM. *n*=8. *P<0.05 versus the Sham+Ad-GFP group; ^#^*P*<0.05 versus the MI+Ad-GFP group.

Cardiac fibrosis was increased in MI rats, which was inhibited by endostatin overexpression (Figure 4A). The increased expressions of collagen I, collagen III, TGF-β, α-SMA, MMP2, and MMP9 in heart of MI rats were reversed by endostatin overexpression (Figure 4B).

**Figure 5:**
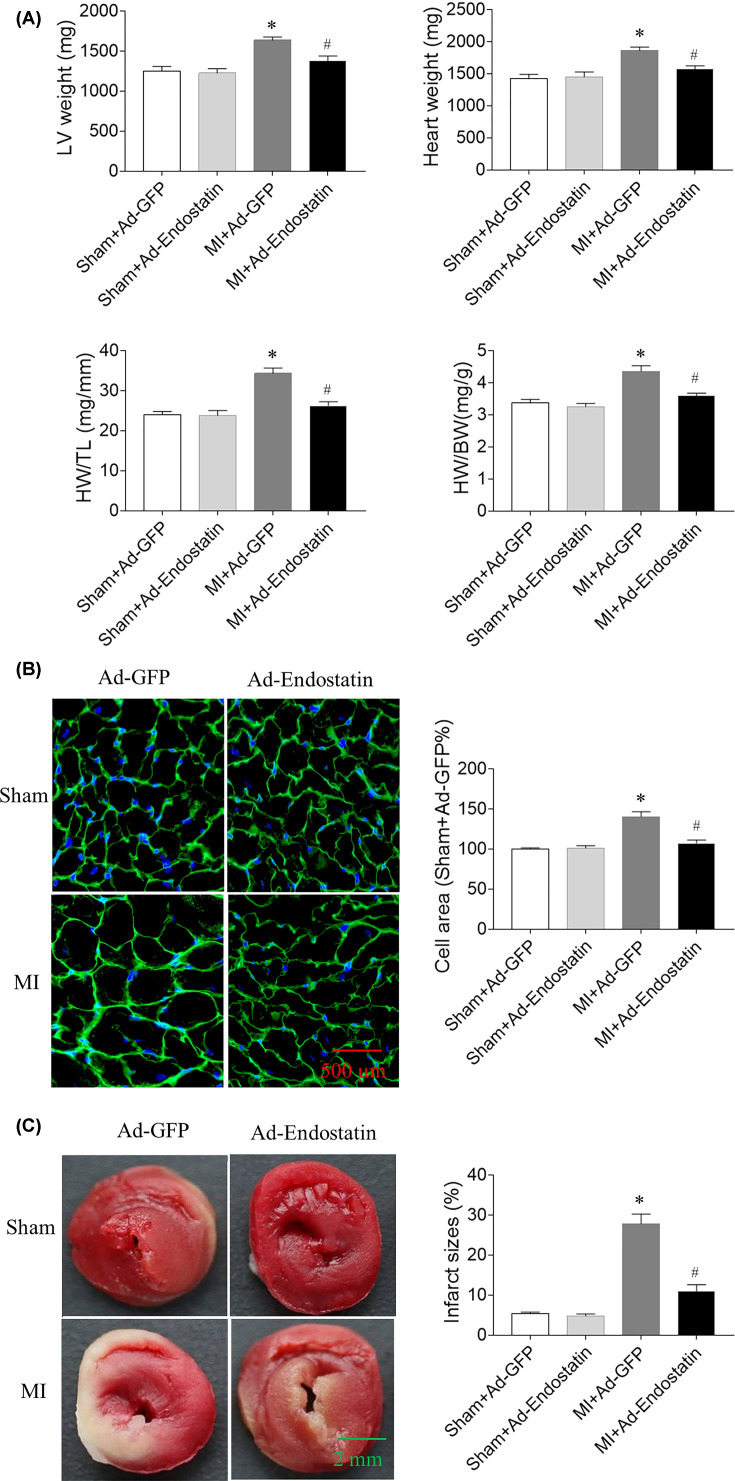
Endostatin attenuated cardiac hypertrophy and infarcted area in MI rats (**A**) Endostatin overexpression attenuated the increase in LV weight, HW, HW/TL, and HW/BW in MI rats. (**B**) Endostatin overexpression attenuated the increase in cardiomyocyte size in MI rats. (**C**) Endostatin overexpression attenuated the increase in infarcted area in MI rats. The results are expressed as mean ± SEM. *n*=8. *P<0.05 versus the Sham+Ad-GFP group; ^#^*P*<0.05 versus the MI+Ad-GFP group.

LV weight, heart weight (HW), HW/body weight (BW), and HW/tibial length (TL) were increased in MI rats, which were reversed by endostatin overexpression (Figure 5A). The size of cardiomyocytes in MI rats was increased, and this increase was attenuated by endostatin overexpression (Figure 5B).

### Effects of endostatin overexpression on infarcted area of MI

The infarcted area of the heart in MI rats was increased, and this increase was inhibited by endostatin overexpression (Figure 5C).

### Levels of MDA, SOD activity, superoxide anion, and Nox activity

**Figure 6:**
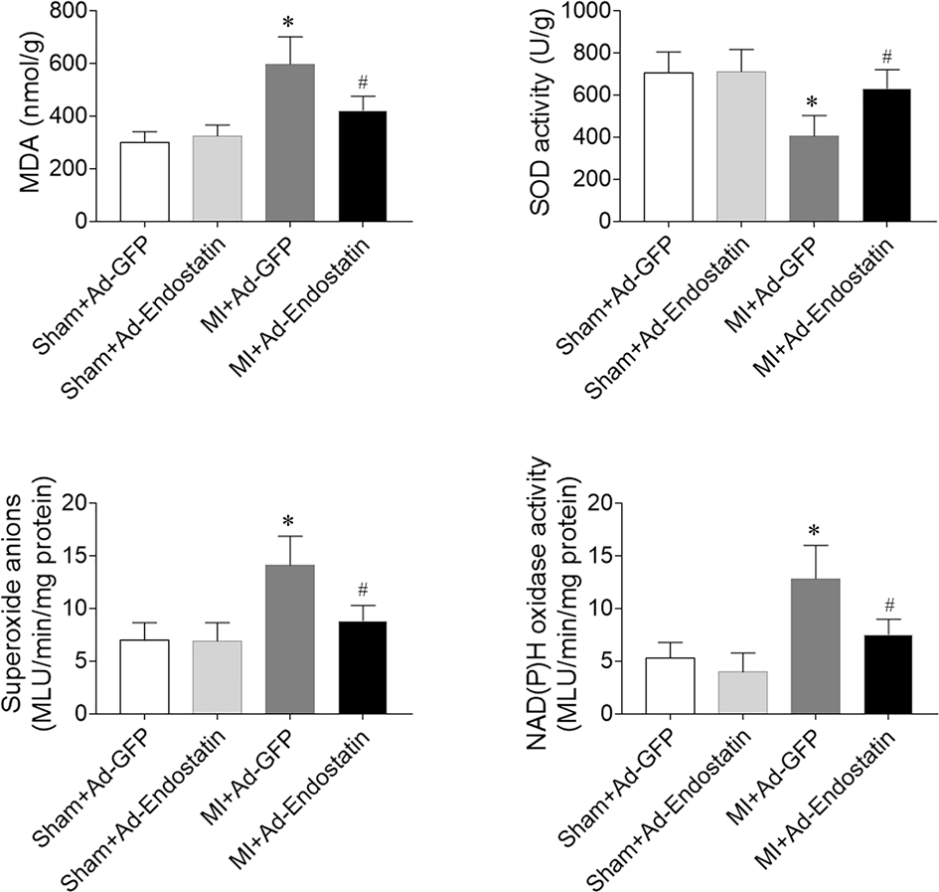
Levels of MDA, SOD activity, superoxide anions, and Nox activity MDA, superoxide anions, and Nox activity levels were increased, and SOD activity level was reduced in the heart of MI rats, which was reversed by endostatin overexpression. The results are expressed as mean ± SEM. *n*=8. *P<0.05 versus the Sham+Ad-GFP group; ^#^*P*<0.05 versus the MI+Ad-GFP group.

The levels of MDA, superoxide anion, and Nox activity were increased in the heart of MI rats, which was reduced by endostatin overexpression. MI attenuated SOD activity in the heart of MI rats, which was reversed by endostatin overexpression (Figure 6).

### Effects of Nox4 overexpression on levels of MDA, SOD activity, superoxide anion, and Nox activity

**Figure 7:**
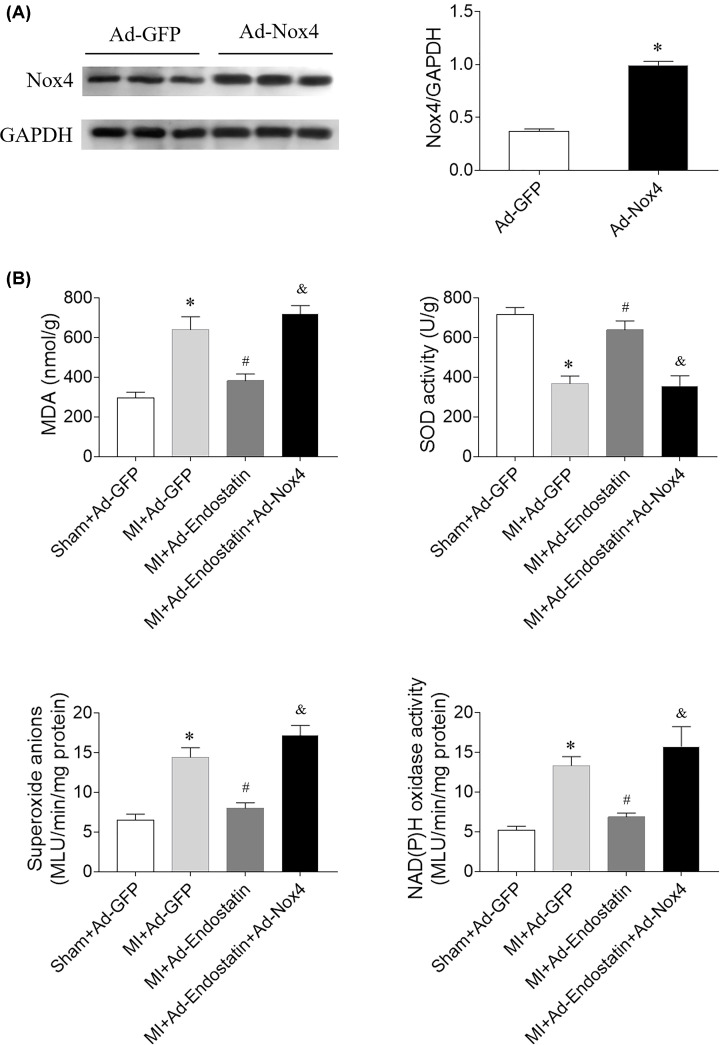
Nox4 overexpression reversed the effects of endostatin overexpression on inhibiting oxidative stresses in the heart of MI rats (**A**) Nox4 expression was increased in the heart of MI rats treatment with Ad-Nox4. (**B**) Nox4 overexpression reversed the effects of endostatin on inhibiting the increases in MDA, superoxide anion and Nox activity, and the decrease in SOD activity. The results are expressed as mean ± SEM. *n*=8. *P<0.05 versus the Ad-GFP (A) or Sham+Ad-GFP (B) group; ^#^*P*<0.05 versus the MI+Ad-GFP group; ^&^*P*<0.05 versus the MI+Ad-Endostatin group.

The expression of Nox4 was increased in the heart of Ad-Nox4 treated rats (Figure 7A). Nox4 overexpression reversed the effects of endostatin on inhibiting the increases in MDA, superoxide anion, and Nox activity, and the decrease in SOD activity (Figure 7B).

### Effects of Nox4 overexpression on endostatin overexpression-induced protective effects on cardiac function

**Figure 8:**
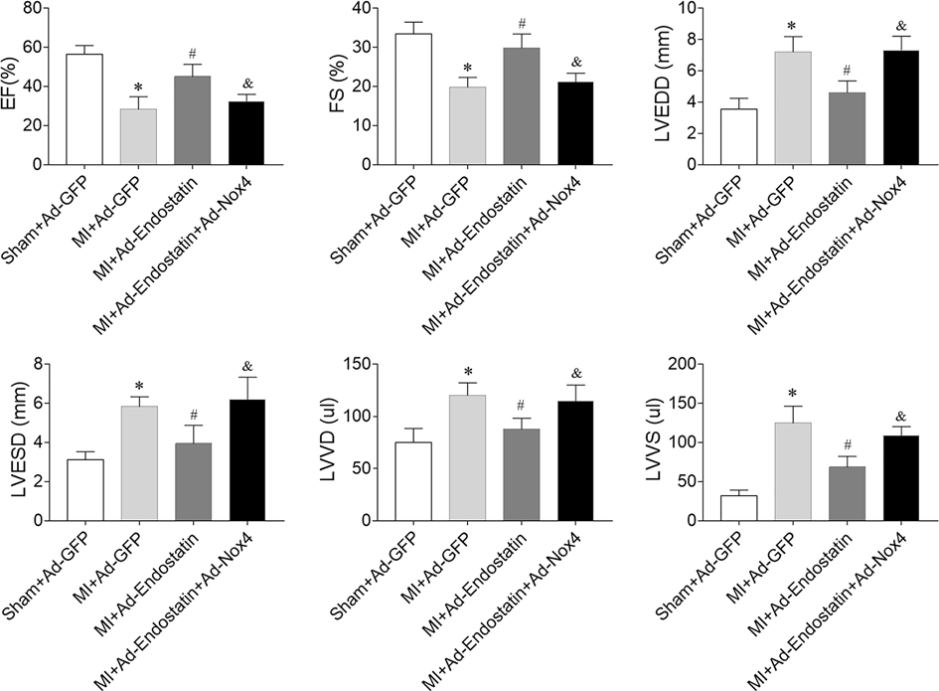
Nox 4 overexpression inhibited endostatin overexpression-induced improvement of cardiac dysfunction in MI rats Nox4 overexpression reversed the endostatin overexpression-induced improvement of LV EF, FS, the increases in LVEDD, LVESD, LVVD, and LVVS in MI rats. The results are expressed as mean ± SEM. *n*=8. *P<0.05 versus the Sham+Ad-GFP group; ^#^*P*<0.05 versus the MI+Ad-GFP group; ^&^*P*<0.05 versus the MI+Ad-Endostatin group.

Endostatin overexpression improved the decreases in EF (%) and FS (%) in MI rats, which was blocked by Nox4 overexpression. Furthermore, Nox4 overexpression reversed endostatin overexpression-induced decreases in LVEDD, LVESD, LVVD, and LVVS (Figure 8).

### Effects of Nox4 overexpression on endostatin overexpression-induced protective effects on cardiac hemodynamics

**Figure 9:**
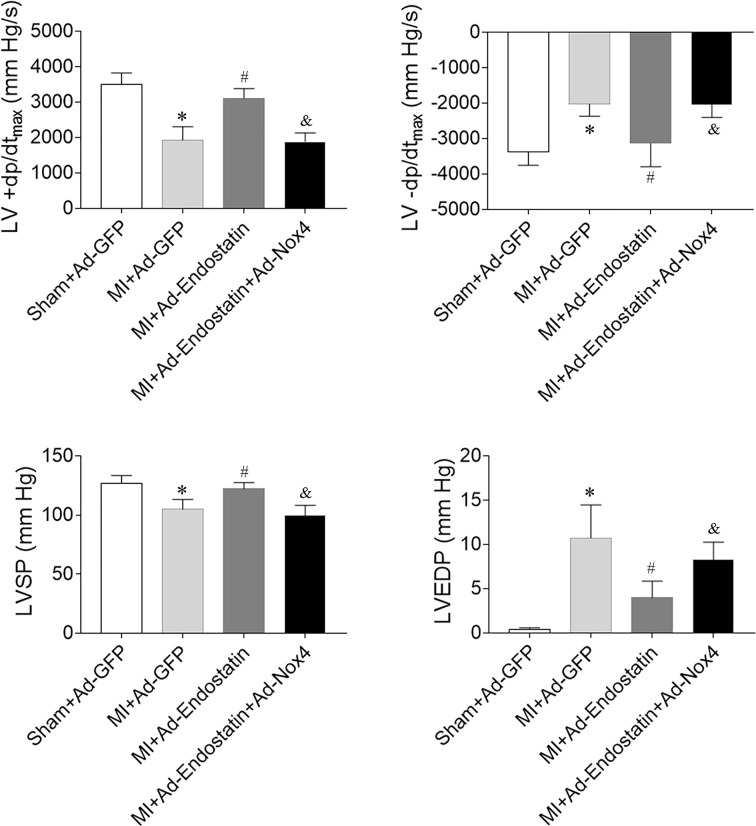
Nox4 overexpression inhibited endostatin overexpression-induced improvement of cardiac hemodynamics in MI rats Nox4 overexpression reversed the effects of endostatin overexpression on improving the maximum of the first differentiation of LV pressure (LV ±dp/dt_max_), LVSP, and LVEDP. The results are expressed as mean ± SEM. *n*=8. *P<0.05 versus the Sham+Ad-GFP group; ^#^*P*<0.05 versus the MI+Ad-GFP group; ^&^*P*<0.05 versus the MI+Ad-Endostatin group.

Endostatin overexpression improved the MI-induced decreases in LV ±dp/dt_max_ and LVSP, which was inhibited by Nox4 overexpression. Endostatin overexpression inhibited the increase in LVEDP in MI rats, which was also reversed by Nox4 overexpression (Figure 9).

### Effects of Nox4 overexpression on endostatin overexpression-induced protective effects on cardiac remodeling

**Figure 10:**
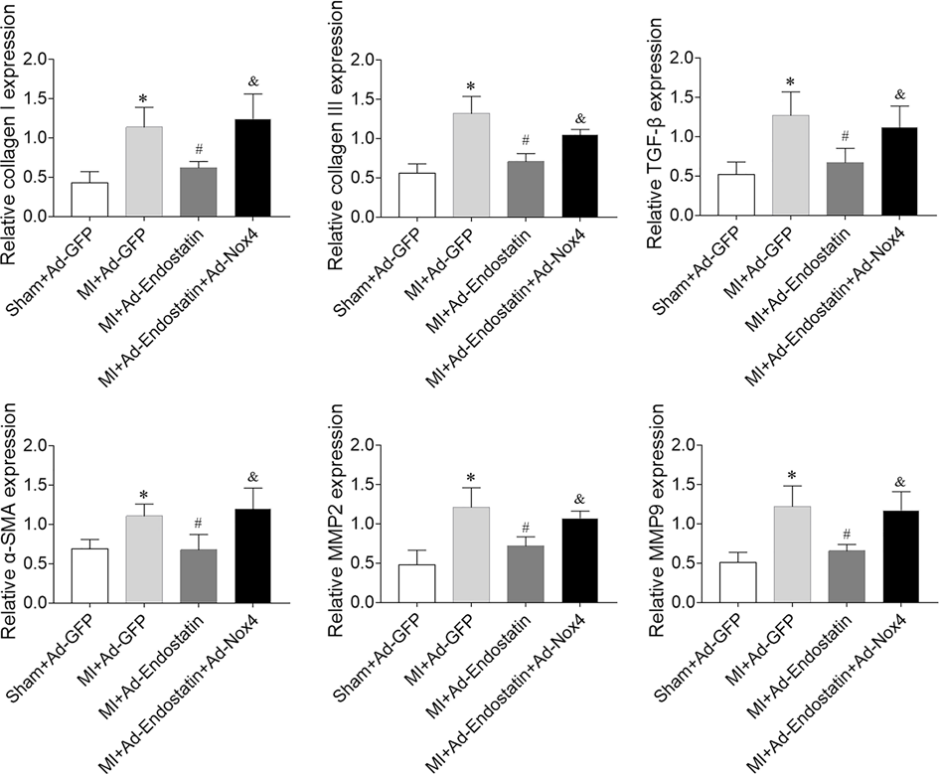
Nox4 overexpression inhibited endostatin overexpression-induced improvement of cardiac fibrosis in MI rats Nox4 overexpression reversed the effects of endostatin overexpression on reducing the levels of collagen I, collagen III, α- SMA, CTGF, MMP-2, and MMP-9 in the heart of MI rats. The results are expressed as mean ± SEM. *n*=8. *P<0.05 versus the Sham+Ad-GFP group; ^#^*P*<0.05 versus the MI+Ad-GFP group; ^&^*P*<0.05 versus the MI+Ad-Endostatin group.

Endostatin overexpression inhibited the increase in collagen I, collagen III, TGF-β, α-SMA, MMP2, and MMP9 in MI rats, which was reversed by Nox4 overexpression (Figure 10).

## Discussion

In many underlying conditions that can lead to HF (such as ischemic heart disease, hypertension, chronic kidney disease, and diabetes), circulating levels of endostatin are elevated. Yet, whether endostatin overexpression attenuates HF and cardiac fibrosis in MI rats are not well known. The present results showed that endostatin overexpression improved cardiac function, hemodynamics, and fibrosis in the heart of MI rats; endostatin attenuated HF via inhibiting oxidative stress.

Endostatin, a C-terminal fragment of collagen XVIIIα1, has a potent anti-angiogenic effect on reducing neointima formation [24]. Endostatin correlates with the severity of diastolic dysfunction and may be a novel biomarker for HF with reduced EF [25]. The results of the present study showed that endostatin level was higher in serum of MI rats. The survival rate was reduced in MI rats compared with the sham group. Endostatin overexpression increased the survival rate of MI rats. These results demonstrated that endostatin may be a therapeutic target for HF.

Cardiac remodeling and cardiac dysfunction are found in chronic HF rats [26]. The cardiac hemodynamics is impaired in chronic HF rats, as manifested by the decreased LVSP and +dp/dt_max_ and increased LVEDP [21]. Endostatin gene knockdown deteriorates monocrotaline-induced right ventricular disease [27]. In the present study, EF (%), FS (%), LV ±dp/dt_max_, LVEDP, and LVSP were reduced in MI rats, which was reversed by endostatin overexpression. LVEDD, LVESD, LVVD, and LVVS were increased in MI rats, and endostatin overexpression inhibited the above-mentioned increases. These results indicated that endostatin improved cardiac dysfunction and the impaired cardiac hemodynamics in MI-induced HF.

MI-induced HF is accompanied by significant cardiac fibrosis [28] and hypertrophy [29]. Endostatin pretreatment can inhibit the fibrosis of human skin fibroblasts and their transformation into myofibroblast [30]. E4 peptide, a peptide derived from endostatin, shows oral bioavailability and exerts anti-fibrotic effects on the bleomycin-induced pulmonary fibrosis mice model [31]. The present study found that the expression levels of collagen I, collagen III, TGF-β, α-SMA, MMP2, and MMP9 were increased in hearts of MI rats, which were reversed by endostatin overexpression. Moreover, LV weight, HW, HW/BW, HW/TL, and cardiomyocytes size were increased, and endostatin overexpression inhibited these increases. The results demonstrated that the MI-induced cardiac fibrosis and hypertrophy were inhibited by endostatin overexpression in MI rats.

ROS are increased in the heart and plasma, and correlate with the severity of LV dysfunction in HF patients [32]. Nox are the major sources of ROS [33–35]. Endostatin stimulates cell proliferation, migration, and wound-induced migration of adult rat cardiac fibroblasts at least partly through the ROS-dependent activation of protein kinase B [20]. The present study showed that increased levels of MDA, superoxide anion, and Nox activity, together with weakened SOD activity in the heart of MI rats, which were reversed by endostatin overexpression. Endostatin overexpression improved the decreased EF (%), FS (%), LV ±dp/dt_max_, and LVSP in MI rats, which was blocked by Nox4 overexpression. Endostatin overexpression inhibited the increases in LVEDP, LVEDD, LVESD, LVVD, and LVVS in MI rats, which were reversed by Nox4 overexpression. Endostatin overexpression inhibited the increase in collagen I, collagen III, TGF-β, α-SMA, MMP2, and MMP9 in MI rats, which were reversed by Nox4 overexpression. These results indicated that endostatin improved cardiac dysfunction, hemodynamics, and cardiac remodeling via inhibiting oxidative stress in MI-induced HF rats.

## Limitations

LV stiffness, one of the earliest parameters to be affected in HF, can be measured with pressure–volume loops [36]. We will determine the effects of endostatin overexpression on LV stiffness with the associated methods in the future. In our present study, we showed that endostatin inhibited the ROS production through inhibiting Nox activity, but the detailed mechanism was not explored. Previous study found that endostatin increases intracellular ceramide levels, which affected ROS production [37].

In conclusion, endostatin overexpression attenuated cardiac dysfunction, impairment of hemodynamics, and cardiac remodeling in HF rats. Endostatin overexpression inhibited the increased oxidative stress, leading to the improvement of HF.

## Abbreviations

Ad-Endostatin: adenoviral vector harboring endostatin
Ad-GFP: adenoviral vector harboring enhanced green fluorescent protein
Ad-Nox4: adenoviral vector harboring NADPH oxidase 4
BW: body weight
CTGF: connective tissue growth factor
EF: ejection fraction
FS: fractional shortening
GAPDH: glyceraldehyde-3-phosphate dehydrogenase
HF: heart failure
HW: heart weight
LV: left ventricle
LVEDD: LV end-diastolic diameter
LVEDP: LV end-diastolic pressure
LVESD: LV end-systolic diameter
LVSP: LV systolic pressure
LVVD: LV volume in diastole
LVVS: LV volume in systole
MDA: malondialdehyde
MI: myocardial infarction
MLU: mean light unit
MMP: matrix metalloproteinase
Nox: NAD(P)H oxidase
ROS: reactive oxygen species
SOD: superoxide dismutase
TU/ml: transducing units per milliliter
α-SMA: α-smooth muscle actin
